# Optimization of the prostaglandin F_2α_ receptor for structural biology

**DOI:** 10.1371/journal.pone.0320114

**Published:** 2025-07-18

**Authors:** Marine Salze, Sébastien Chrétien, Tegvir S. Boora, Madalina Macovei, Eric Barbeau, Véronique Blais, Stéphane A. Laporte, Martin Audet

**Affiliations:** 1 Department of Pharmacology and Physiology, Faculty of Medicine and Health Sciences, Université de Sherbrooke, Sherbrooke, Québec, Canada; 2 Division of Clinical and Translational Research, Department of Medicine, McGill University, Montréal, Québec, Canada; 3 Department of Medicine, McGill University Health Center, McGill University, Montréal, Québec, Canada; 4 Department of Pharmacology and Therapeutics, McGill University, Montréal, Québec, Canada; 5 Centre de Recherche du CHUS, CIUSSS-Estrie-CHUS, Sherbrooke, Québec, Canada; University of the Pacific, UNITED STATES OF AMERICA

## Abstract

Prostaglandin F_2ɑ_ (PGF_2ɑ_) is a bioactive lipid derived from arachidonic acid and is involved in many physiological and pathophysiological processes, such as parturition, vascular tone regulation, glaucoma and inflammation. It acts by binding to the Prostaglandin F_2ɑ_ receptor (FP), a G Protein-Coupled Receptor (GPCR) that mediates signaling events by engaging intracellular heterotrimeric G protein effectors. The orthosteric binding site of lipid-binding receptors displays greater efficacy-dependent plasticity that hinders the design of ligands. Solving the structure of FP with ligands of different efficacies at an atomic level is important to fully understand its mechanism of activation and inhibition. Most purified FP-ligand complexes are unstable *in vitro*. The development of new X-ray crystallography and single particle cryo-electron microscopy (cryoEM) strategies to understand receptors’ signal transduction requires improved purification yield and *in vitro* stability of the receptor. Here, we present a protein engineering effort to optimize the FP protein sequence for use in structural biology. Strategies involve protein insertion sites in the third intracellular loop (ICL3), N-terminal and C-terminal deletions, and single-point mutations that favorably affect receptor purification yield and stability *in vitro*. The best FP construct displays a yield of 1.5 mg/L and a stability of 59^o^C, which constitute a threefold improvement in purification yield and 9^o^C increase in stability over the wild-type receptor. These modifications in the receptor are suitable for pursuing alternative strategies for improving FP purification yield and for studying FP binding efficacy to its ligands through structural biology approaches.

## Introduction

G Protein-Coupled Receptors (GPCRs) form the largest family of membrane proteins in the human genome and are the target of more than 35% of drugs in Western countries [1,2]. Prostaglandin F_2α_ (PGF_2α_) is a bioactive lipid that binds and activates Prostaglandin F_2α_ receptor (FP), a GPCR at the cellular plasma membrane, leading to the engagement of intracellular heterotrimeric G proteins effectors and subsequent activation of cellular signaling cascades [3,4]. PGF_2α_-mediated activation of FP plays an important role in many physiological processes and pathological conditions such as parturition, vascular tone regulation, intracerebral hemorrhage, atherosclerosis, ocular hypertension and glaucoma [4–9], making it an important pharmaceutical target. As such, PGF_2α_ derivatives are currently used in clinics. For example, FP activators (agonists) such as fluprostenol and latanoprost are used for the treatment of glaucoma and ocular hypertension [4]. Cloprostenol, the most potent FP ligand, is used for labor induction [4]. Few FP inhibitors (antagonists) have been developed, such as AL8810 [10], PDC113.824 (a positive allosteric modulator biased toward the Gq pathway) [11], BAY-6672 [9], and OBE022, which showed encouraging outcomes in clinical trials for addressing preterm labor [12]. Overall, the development of antagonists targeting FP remains a challenge [13].

Recently, the development of new approaches in structural biology has led to high-resolution structures of FP-bound PGF_2α_ and closely related ligands, providing the first insight into agonist binding to the receptor [14,15]. In these structures, FP shares the typical GPCR topology, consisting of 7 transmembrane helical domains, 3 intracellular loops, 3 extracellular loops, a helix 8 parallel to the membrane, and extended N- and C-terminal tails. The receptor folds as a 7 transmembrane helical bundle with PGF_2α_ binding at the orthosteric site, inside the bundle, covered by an extended N-terminus that serves as a lid to the receptor. This feature, shared by many lipid binding receptors, contributes to the typical low off-rate of lipid receptor ligands and to the difficulty of determining their pharmacological profiles [14–17]. In addition, the greater efficacy-dependent plasticity of lipid receptor binding sites complicates the rational design of antagonists [16]. A full understanding of the mechanisms of activation and inhibition of FP requires solving structures with ligands of different efficacies.

Stabilization and improvement of the purification yield of GPCRs through optimization of receptor sequences have been key features in the recent development of GPCR structural biology derived from cryo-electron microscopy (cryoEM) and X-ray crystallography approaches [18–20]. So far, FP has only been stabilized by the binding of an agonist and in complex with a chimeric heterotrimeric G protein and stabilizing nanobody [14,15]. In addition, FP has never been crystallized, and apo or antagonist-bound receptors are unstable *in vitro.* This difficulty is shared among some of the prostaglandin receptors, as there is only one co-crystal structure with an antagonist, the PGE_2_ type 4 receptor bound to ONO-AE3-208 [21]. This structure required extensive receptor optimization and the use of a Fab antibody to stabilize the antagonist-bound conformation of the receptor. To foster further structural studies of FP and gain a full understanding of the ligand’s mechanism of efficacy, it is imperative to improve methods to overcome its lack of stability during purification*.* Here, we present work on the optimization of the receptor protein sequence to improve the purification yield and stability of the FP receptor *in vitro*.

## Materials and methods

### Cloning and gene modification

Human FP gene sequence was codon optimized for *Sf9* and human cell expression, synthesized by GeneScript (Piscataway, USA) and cloned into the pFastBac® vector with a FLAG tag, 10xHis tag and TEV cleavage site at the N-terminus. Insertion of soluble domains, receptor truncation and single residue mutations were performed by PCR using Phusion® polymerase (ThermoFisher Scientific, Waltham, USA). For expression in HEK293T cells, the tagged receptor constructs were subcloned into pcDNA3.1(-) using BamHI-HF/HindIII-HF and T4DNA ligase (New England Biolabs).

### Expression and purification of FP

FP constructs were expressed in *Sf9* insect cells using the Bac-To-Bac Baculovirus expression system (ThermoFisher Scientific). *Sf9* insect cell line is a clonal isolate derived from the parental *Spodoptera frugiperda* cell line IPLB-Sf-21-AE purchased directly from Expression Systems (cat#94-001F). They were grown in ESF 921 Insect cell culture protein free media (Expression Systems, Davis, USA) at 27°C with shaking. Membranes from cells expressing FP constructs were prepared using two rounds of washing and centrifugation at 45,000 X g, first in the presence of a lysis buffer containing 10 mM HEPES, pH 7.5, 20 mM KCl, and 10 mM MgCl_2_, and then with a washing buffer containing 10 mM HEPES pH 7.5, 1 M NaCl, 20 mM KCl and 10 mM MgCl_2_. Protease inhibitors (1 mM Benzamidine and 1 µg/mL leupeptin) were added in both steps. The purified membrane was then resuspended in a storage buffer (10 mM HEPES pH 7.5, 20 mM KCl, 10 mM MgCl_2_, 20% (v/v) Glycerol). Membranes containing receptors were then solubilized in 50 mM HEPES pH 7.5, 800 mM NaCl, 5 mM KCl, 2.5 mM MgCl_2_, 0.5% (w/v) n-dodecyl-β-D-maltopyranoside (DDM, Anatrace), and 0.1% (w/v) cholesteryl hemisuccinate (CHS, Sigma) in the presence of protease inhibitors, 1 mg/mL iodoacetamide and 10 µM cloprostenol (Cayman chemical, USA). The supernatant was isolated by centrifugation for 1 h at 21,000 X g and then incubated with TALON resin (TALON® Superflow™ beads, Cytiva) overnight at 4^o^C. The TALON resin was washed with 20 column volumes of wash buffer 1 containing 50 mM HEPES pH 7.5, 150 mM NaCl, 20 mM MgCl_2_, 20 mM imidazole, 8 mM ATP, 10% (v/v) glycerol, 0.1% (w/v) DDM, 0.02% (w/v) CHS, and 1 µM cloprostenol, followed by 10 column volumes of wash buffer 2 containing 50 mM HEPES pH 7.5, 150 mM NaCl, 30 mM imidazole, 10% (v/v) glycerol, 0.05% (w/v) DDM, 0.01% (w/v) CHS, and 1 µM cloprostenol. The receptor was eluted using 6 column volumes of elution buffer containing 50 mM HEPES pH 7.5, 150 mM NaCl, 250 mM imidazole, 10% (v/v) glycerol, 0.015% (w/v) DDM, 0.003% (w/v) CHS, and 1 µM cloprostenol. Finally, the purified receptor eluate was concentrated using a vivaspin 500, 100 Kd (Sartorius), and analyzed by size exclusion chromatography (SEC) on an Infinity II HPLC system (Agilent) using an SRT-C SEC-300 column (Sepax). Purification yield was determined using UV_280_ absorbance peak at the elution time corresponding to the monodispersed state of the receptor on HPLC-SEC chromatogram.

### Microscale fluorescence thermal assay

7-Diethylamino-3-(4’-Maleimidylphenyl)-4-Methylcoumarin (CPM) dye was obtained from ThermoScientific and resuspended in DMSO at a concentration of 500 µM. 2 µg of purified protein was added to the reaction buffer (50 mM HEPES pH 7.5, 150 mM NaCl, 0.05% (w/v) DDM, 0.01% (w/v) CHS, and 5 µM CPM). The samples were then treated with 100 µM cloprostenol or vehicle and incubated for 20 min on ice. The unfolding of FP was induced using a Rotor-Gene Q (QIAGEN) thermocycler by slowly increasing the sample temperature (+0.5^o^C/ step from 30°C to 95°C), resulting in an emission of a fluorescence signal detected in the blue channel (excitation 365nm/emission 460nm) from the CPM chemical reaction to newly exposed receptor cysteines. The melting temperature (Tm) was determined as the temperature at the maximal value of the first derivative of the fluorescence vs temperature thermodenaturation curve.

### Cell signaling assay

HEK293T cells (ATCC, cat# CRL-3216) were cultured in Dulbecco’s Modified Eagle Medium (DMEM) supplemented with 10% fetal bovine serum (FBS) and 20 μg/mL gentamicin at 37°C in 5% CO₂ and 90% humidity. Cells were seeded in white, flat-bottom 96-well plates at a density of 9,000 cells per well and transiently transfected 24 hours later with 150 ng of FP receptor DNA, 100 ng of RhoA BRET biosensor DNA, and 750 ng of empty pcDNA3.1 vector to reach a total of 1 µg DNA per well. A previously described bioluminescence resonance energy transfer (BRET)-based biosensor was used to measure G protein signaling via RhoA activation [22,23]. The biosensor detects RhoA translocation to the plasma membrane, a downstream event of G protein activation. It consists of a Rho-binding domain (RBD) of protein kinase N (PKN) fused to Renilla luciferase (PKN-RBD-RlucII) as the donor, and the acceptor protein rGF) anchored to the plasma membrane via a CAAX motif (rGFP-CAAX). Upon RhoA activation, PKN-RBD is recruited to the membrane to the endogenous G protein, bringing RlucII into proximity with membrane-bound rGFP, allowing bystander BRET. This spatial interaction enables energy transfer from RlucII to rGFP following the addition of the substrate, coelenterazine. To initiate the BRET reaction, 2.5 μM coelenterazine 400a (GoldBio), a cell-permeable RlucII substrate, was added 3.5 minutes prior to measurement. Coelenterazine oxidation by RlucII produces light at ~410 nm (donor emission), which excites rGFP when the donor and acceptor are sufficiently close (within 10 nm), leading to emission at ~515 nm that is in the range of the acceptor rGFP excitability. BRET ratios were calculated as the emission at 515 nm divided by the emission at 410 nm. All assays were performed 48 hours post-transfection. On the day of the experiment, cells were incubated in Tyrode’s buffer (25 mM HEPES, pH 7.4, 140 mM NaCl, 12 mM NaHCO₃, 5.6 mM D-glucose, 2.7 mM KCl, 1 mM CaCl₂, 0.5 mM MgCl₂, 0.37 mM NaH₂PO₄) for 1 hour. Cells were then stimulated with serial dilutions of PGF_2α_ or cloprostenol (10^-10.5^ to 10^−5^ M), and BRET signals were recorded using a BioTek Synergy 2 plate reader equipped with 410/80 nm (donor) and 515/30 nm (acceptor) filters. Data were fitted to 12-point concentration–response curves and analyzed for extracting pharmacological parameters.

### Data presentation and statistical analysis

**Fig 1:**
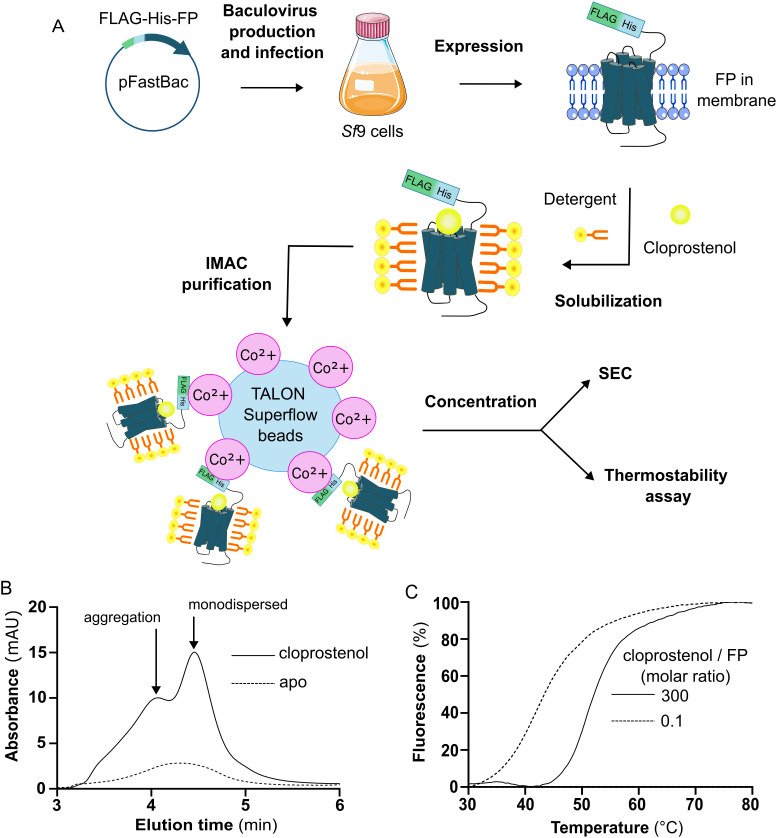
Purification of FP in complexes with cloprostenol. **(A)** Cartoon of the steps leading to FP purification by IMAC and characterization using HPLC-SEC and microscale fluorescence thermal assay. **(B)** Representative HPLC-SEC chromatogram of purified FP-WT (Wild Type) in the presence and absence of 100 μM of cloprostenol. The mean ± SEM of FP purification yield determined from 3 independent experiments is 0.097 ± 0.008 and 0.25 ± 0.02 mg/ L for the apo and the cloprostenol-bound forms, respectively. **(C)** Representative thermostability curve of FP-cloprostenol complex at cloprostenol/ FP molar ratio of 300 (100 μM of cloprostenol) and 0.1 (0.02 μM of cloprostenol). The mean ± SEM of the Tm value determined from 3 independent experiments is 50.8 ± 0.4^o^C and 42.6 ± 0.3^o^C at molar ratios of 300 and 0.1, respectively.

Graphs were created using Graphpad Prism software. Cartoons in Fig 1 were derived from the bioicons database. Snake-plots were generated by the GPCR database (GPCRdb). Figures were mounted using Inkscape software. Statistical analyses were performed using Graphpad Prism software. Statistical significance was determined by a one-way ANOVA followed by a Dunnett post-hoc analysis.

## Results

### FP purification and stability

To assess the stability of FP in micelles, we first inserted the coding sequence of the receptor fused with a FLAG and a polyhistidine (10xHis) affinity tags at the N-terminus into the pFastBac1 vector. This vector is used to produce baculoviruses that express the FP coding sequence in *Sf*9 insect cells (Fig 1A). We then purified FP from a membrane preparation of these cells using immobilized metal affinity chromatography (IMAC) and analyzed the purification yield and aggregation state of the receptor in DDM/CHS micelles using size exclusion chromatography by HPLC (HPLC-SEC) (Fig 1B). The apo form of FP displayed a low purification yield of 0.097 mg/L and a wide peak on the chromatogram with an elution time of 4.1 minutes, corresponding to a higher order aggregation state of the receptor. Since agonist-induced receptor stabilization *in vitro* has already been observed for another prostaglandin receptor and other GPCRs [17,24], we added a saturating amount of cloprostenol, a stable PGF_2α_ analog, at all steps of FP purification. This resulted in the appearance of a major elution peak at 4.5 minutes and a minor peak at 4.0 mins, corresponding to the monodispersed and aggregated form of the receptor, respectively. Overall, the monodispersed form of the FP-cloprostenol complex showed a 2.5-fold increase in the purification yield compared to the FP apo aggregated form (0.247 mg/L vs 0.097 mg/L, respectively). We further measured the cloprostenol-induced stability of the receptor using a microscale thermal stability assay on the FP-cloprostenol purified complex (Fig 1C). Upon heat-induced denaturation, proteins expose embedded cysteine residues that react with the CPM dye, leading to fluorescence [25]. The inflection point of the thermodenaturation curve (fluorescence vs. heat) corresponds to the melting temperature (Tm) of the receptor, providing a direct measure of its stability. To establish a baseline for the effect of cloprostenol on FP stability, given that the apo form could not be purified, we measured the Tm at a freshly ligand-depleted cloprostenol/ FP molar ratio of 0.1 (Fig 1C; S2 Fig) and obtained a Tm of 43^o^C. In contrast, FP-cloprostenol complex at a saturating cloprostenol/ FP molar ratio of 300 exhibited a Tm of 50^o^C. Although this represents a clear stabilization upon ligand binding, the Tm remains below the threshold of 60°C typically required for structural biology applications, highlighting the need for additional optimization. Given the positive impact of cloprostenol on FP purification yield and stability, we included the ligand for all subsequent purification steps described in this study. Our objective is to further enhance the stability and purification yield of the FP-cloprostenol complex through targeted FP protein sequence optimization.

### Stabilization of FP by bRIL insertion

**Fig 2:**
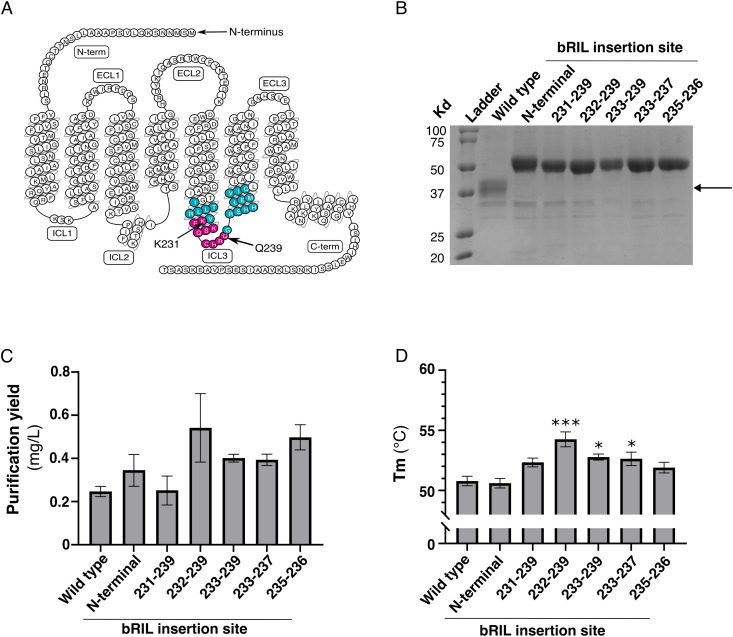
Impact of bRIL domain fusion on the stability of FP. **(A)** Snake plot of FP. Predicted ICL3 is indicated in blue and magenta circles. bRIL insertion in ICL3 was screened between residues 231 and 239 of FP as shown in magenta circles. **(B)** Coomassie-stained 12% polyacrylamide gel of the IMAC eluate of the indicated FP-bRIL fusions. The arrow indicates the expected migration of endogenous insect cell heterotrimeric Gα subunit. **(C)**. Purification yield of bRIL fusion at the FP N-terminal, or between the residues indicated, in complex with cloprostenol. **(D)** Melting temperature (Tm) of the indicated FP fusions. The data are expressed as the means ± SEM of 3 independent experiments. Statistical significance was determined by one-way ANOVA followed by a Dunnett post-hoc analysis. * p-value < 0.05; *** p-value < 0.001.

Insertion of small soluble domains into GPCR intracellular loops has already been used to help their stabilization and to improve the purification yield [18,20]. Thus, we inserted the soluble apocytochrome b562RIL (bRIL) from *E. coli* at the N-terminus of FP and into different positions of the predicted third intracellular loop (ICL3) [18] from residues 231–239 (Fig 2A), and determined the receptor purification yield and the stability in DDM micelles using the microscale thermal fluorescence assay (Fig 2B–2D). All FP constructs were successfully expressed in *Sf*9 cells (S3 Table) and purified to at least 95% purity, as shown by a Coomassie-stained protein gel of the receptors (Fig 2B). The insertion of bRIL increased the purification yield of FP for all the constructs, except the fusion between residues 231–239. The construct with the insertion between residues 232–239 showed the highest purification yield, achieving a two-fold improvement in the purification yield of 0.542 mg/L compared to 0.247 mg/L for FP without bRIL insertion. Similarly, all but one construct displayed at least a noticeable increase in stability, with three out of six being statistically significant improvements. Insertion of bRIL at position 232–239 showed the best improvement in stability with an increased Tm of 4^o^C (from 50 to 54), as compared to FP without insertion. N-terminal bRIL fusion to FP slightly improved the purification yield but not the stability of FP, whereas ICL3 bRIL fusion tended to improve both purification yield and stability, except for the construct 231–239 that only showed an apparent but not significant increase in Tm. Interestingly, no bands corresponding to endogenous heterotrimeric G protein subunits could be found on the gel (Fig 2B), indicating that coupling to endogenous effectors does not contribute to cloprostenol-induced receptor stabilization *in vitro*. Overall, the insertion of bRIL between residues 232–239 of ICL3 resulted in the best improvement in both purification yield and stability of FP in micelles.

### Screening of N- and C-termini deletions

**Fig 3:**
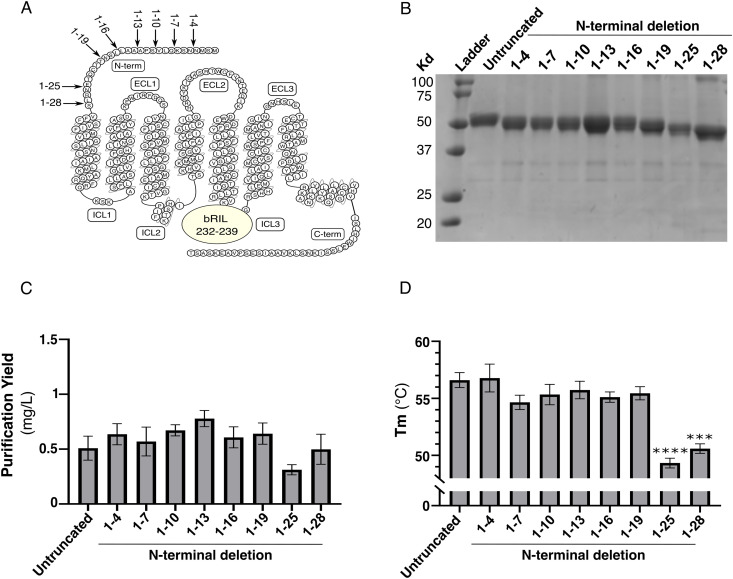
Screening of N-terminal deletion on FP stability. **(A)** Snake plot of FP. N-terminal deletions of the different constructs are shown by arrows. bRIL insertion in ICL3 between residues 232 and 239 is represented by a circle. **(B)** Coomassie-stained 12% polyacrylamide gel of the IMAC eluate of the indicated FP N-terminal deletions. **(C)** Purification yield and (D) melting temperature (Tm) of the IMAC eluate of indicated FP N-terminal deletions. The untruncated receptor is FP in fusion with bRIL between the receptor residues 232-239 of ICL3. The range indicates the N-terminal domain deleted from the untruncated receptor. The data are expressed as the means ± SEM of 3 independent experiments. Statistical significance was determined by one-way ANOVA followed by a Dunnett post-hoc analysis. *** p-value < 0.001; **** p-value < 0.0001.

We took advantage of the bRIL 232–239 insertion to further assess the impact of N-terminal truncation on FP purification yield and stability (Fig 3). The N-terminus of FP with the bRIL 232–239 insertion was incrementally deleted from residues 4–28, corresponding to the predicted span of the N-terminal domain in the FP cryoEM structure [14] (Fig 3A). Each construct was successfully expressed in *Sf*9 insect cells (S3 Table) and the IMAC elution fractions displayed a purity of at least 95% (Fig 3B). Deletion of fragments of the FP N-terminus generally did not affect the purification yield or the stability of FP compared to the untruncated construct (Fig 3C, 3D) until the truncation of residue 25, which corresponds to four residues before the beginning of transmembrane helix 1 in the FP cryoEM structure [14]. This suggests that transmembrane helix 1 may begin to form a helical turn earlier than indicated in the assigned structure, or that the few residues at the N-terminus preceding transmembrane helix 1 play a critical role in receptor folding. Overall, truncation of the N-terminus is well tolerated in *Sf*9 cells but does not provide improvement in purification yield or stability. These findings are useful in structural biology because they allow reducing the risk of flexible domain-induced receptor aggregation and interference with crystal contact.

**Fig 4:**
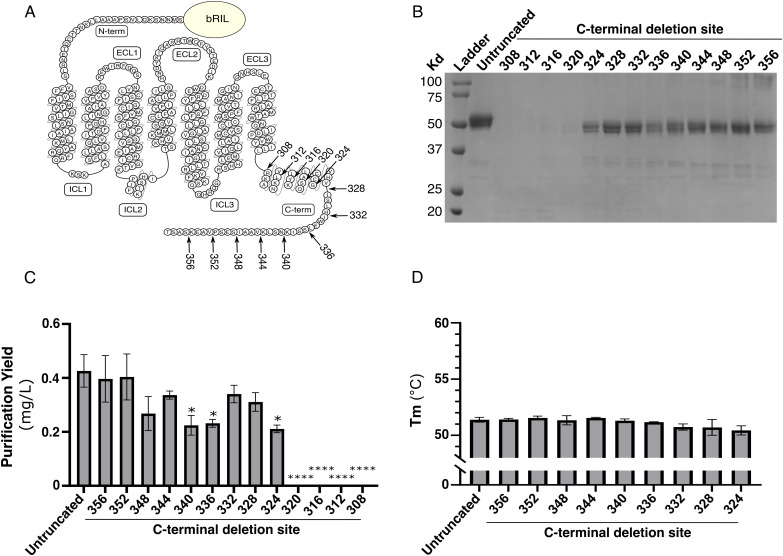
Screening of C-terminal deletion on FP purification yield. **(A)** Snake plot of FP. C-terminal deletion on the different constructs is shown by arrows. bRIL insertion at the N-terminus is represented by a circle. **(B)** Coomassie-stained 12% polyacrylamide gel of the IMAC eluates, **(C)** Purification yield and (D) melting temperature (Tm) of the indicated receptor C-terminal deletions. The untruncated receptor is an N-terminal fusion of bRIL with FP. The numbers indicate the last residue truncated from the C-terminus of the receptor. The data are expressed as the means ± SEM of 3 independent experiments. * p < 0.05; **** p < 0.0001.

In parallel, we investigate the effect of the C-terminal truncation on receptor purification yield and stability in the context of the N-terminal fusion of bRIL with FP (Fig 4). To achieve this, we truncated the C-terminus of the receptor every four residues up to residue 308, which corresponds to the beginning of helix 8 in the FP cryoEM structure [14] (Fig 4A). All truncated constructs expressed well in *Sf*9 cells (S3 Table). We could purify all the FP constructs up to the truncation at residue 324 (Fig 4B, 4C). Further truncations to residues 320, 316, 312 and 308, although well expressed in cells, could not be purified. Overall, our findings imply that truncation of the C-terminal domain does not improve the purification yield and does not affect FP stability, at least for the purified truncations up to residue 324 (Fig 4D).

### Insertion of mutations

**Fig 5:**
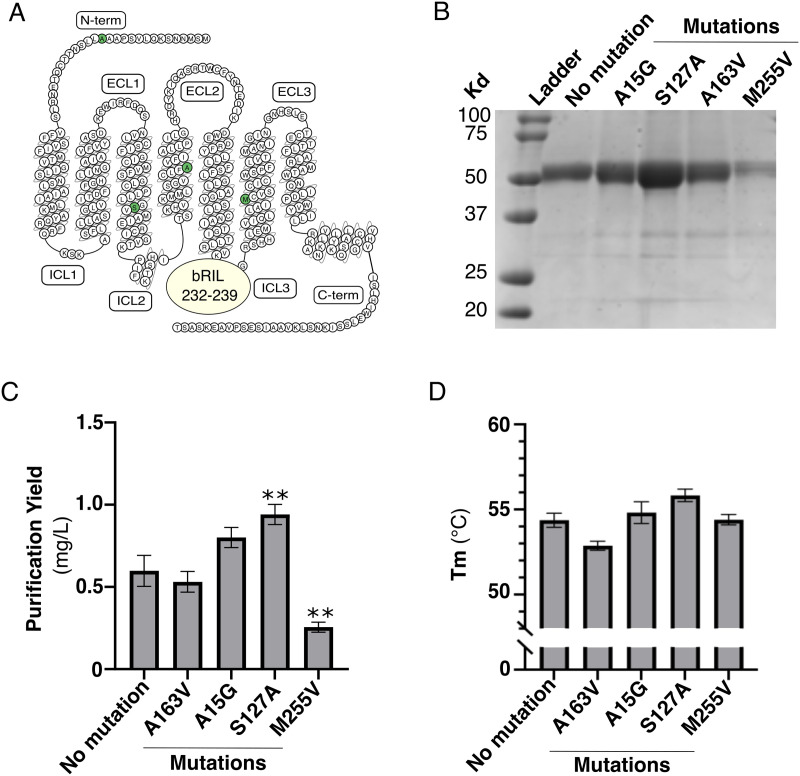
Assessment of predicted stabilizing mutations on FP. **(A)** Snake plot of FP. Mutations are indicated by green circles. bRIL insertion in FP ICL3 between residues 232 and 239 is shown by a circle. **(B)** Coomassie-stained 12% polyacrylamide gel, (C) purification yield, and (D) melting temperature (Tm) of the IMAC eluate of the indicated FP constructs. The parental construct is described in Fig 3. The data are expressed as the means ± SEM of 3 independent experiments. Statistical significance was determined by one-way ANOVA followed by a Dunnett post-hoc analysis. ** p < 0.01.

**Fig 6:**
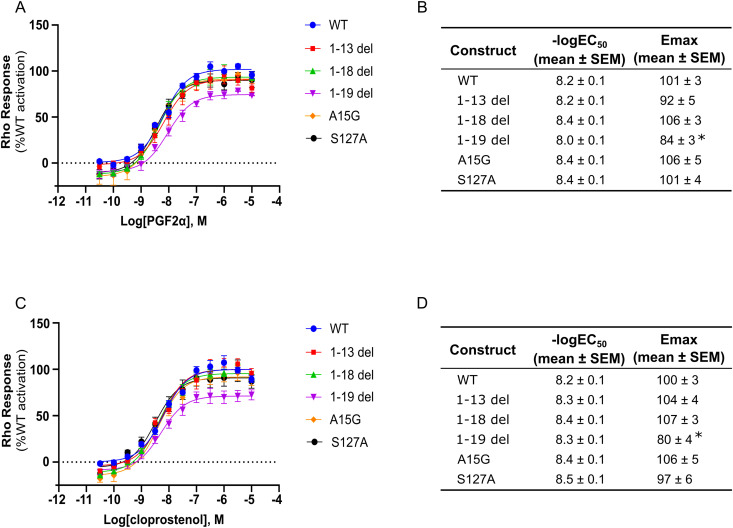
Functional impact of FP modifications. Dose-response curves of **A)** PGF_2α_ and C) cloprostenol-induced activation of the Rho signaling pathway via FP. WT and mutant FP receptors were transiently expressed in HEK293T cells along with the RhoA BRET biosensor to measure G protein response following PGF_2ɑ_ and cloprostenol stimulation, respectively. BRET signals were normalized to the maximal response of WT and plotted as 12-point dose-response curves using GraphPad Prism. The tables present the -logEC_50_ and Emax derived from **B)** PGF_2α_ and D) cloprostenol-induced FP activation. The data are expressed as the mean ± SEM of A-B) 4 and C-D) 5 independent experiments. Statistical significance was determined using one-way ANOVA followed by Dunnett post-hoc analysis. * p < 0.05.

The insertion of stabilizing mutations has been key to improving GPCR recovery during purification [26]. We thus further assessed the impact of single residue mutations on receptor stability and purification yield. We tested three mutations, A15G, S127A, and M255V, inserted into a bRIL fusion at position 232–239 of FP (Fig 5A). A15G was selected, as it was previously found that it increased receptor cell expression in human embryonic kidney cells (Personal communication from Stephane Laporte, 2021). Alanine substitution at residue 127 aimed to reduce hydrophilic residue content in the membrane facing pocket formed by transmembrane helices 3-4-5, and is a similar previously used strategy that further stabilizes the crystal structure of the ß_2_-adrenergic receptor in a complex with an inverse agonist [27]. Finally, M255V was selected because it is a stabilizing mutation predicted by GPCRdb [26]. As a control, we inserted a valine at position 163 of FP, a position homologous to the V185 in the PGE_2_ type 3 receptor (EP3). In the EP3-PGE_2_ co-crystal structure, the valine at position 185 was mutated to a serine to stabilize the receptor [28]. Thus, alanine substitution of a valine at position 163 of FP would not be predicted to improve the receptor stability. Coomassie-stained protein gel shows that we successfully purified all mutations by IMAC to the purity of 95% (Fig 5B). Among all mutations, we found that the control A163V and M255V did not improve the purification yield or stability of FP (Fig 5C, 5D). In contrast, S127A exhibited a significant increase in purification yield along with a slight apparent improvement in stability, whereas A15G showed a minor apparent increase in purification yield but it did not affect receptor stability in agreement with our predictions. Overall, both S127A and A15G were found to enhance the purification outcome of FP, albeit to a different extent. We next sought to assess the impact of our mutants and deletions on FP signaling using a Rho-based BRET cellular assay that captures all cognate G protein coupling of the receptor, including G_12/13_ [3]. The dose-response curves of PGF_2α_ and cloprostenol in HEK293T cells overexpressing FP receptors with N-terminal deletions at positions 13, 18 and 19, as well as A15G and S127A mutants, yielded EC_50_ values that were not statistically different from those observed with the wild-type receptor. Interestingly, while most modifications do not affect Emax, the FP receptor with an N-terminal deletion at position 19 exhibits a modest but statistically significant reduction in Emax compared to the WT receptor upon stimulation with both PGF_2α_ and cloprostenol. This suggests that, in HEK293T cells, the deletion at position 19 may slightly impair the receptor’s signal transduction efficacy. Overall, these findings indicate that the N-terminal deletions and the A15G and S127A mutations do not affect the receptor’s function with respect to the ability of the receptor to engage and respond to agonist stimulation (Fig 6).

### Screening of small soluble domains and combination of most stable modifications

**Fig 7:**
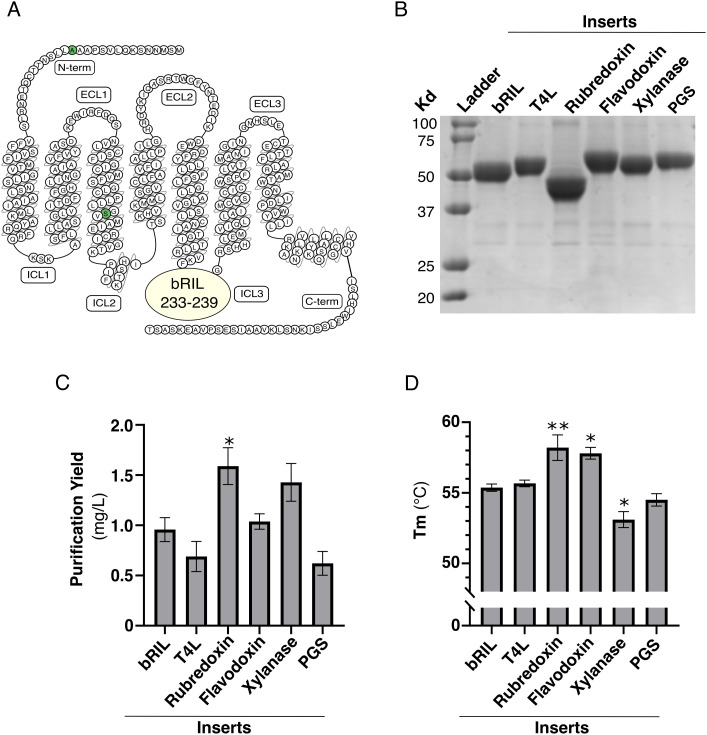
Impact of soluble domain on FP. **(A)** Snake plot of FP. FP fusions have the indicated small domain inserted between residues 233-239 of the ICL3, and harbor stabilizing mutations A15G and S127A (green circles). **(B)** Coomassie-stained 12% polyacrylamide gel, (C) purification yield and (D) melting temperature (Tm) of the IMAC eluate of the indicated FP fusions with different soluble domains. The data are expressed as the means ± SEM of 3 independent experiments. Statistical significance was determined by one-way ANOVA followed by a Dunnett post-hoc analysis. * p < 0.05; ** p < 0.01.

Our previous screening of bRIL fusion within the FP ICL3 allowed us to identify a few key insertion sites for small soluble domains that improve the purification yield and stability of FP-WT (Fig 2). To evaluate the potential benefit of the fusion of other small soluble domains previously used to stabilize GPCRs, we inserted T4 lysozyme (T4L), the thermostable glycogen synthase domain from *Pyrococcus abyssi* (PGS), rubredoxin, flavodoxin or xylanase between residues 233–239 of FP (Fig 7A). We also included the favorable mutations A15G and S127A in all FP fusions [18,20,29]. The new constructs were successfully expressed, and FP-fusion eluates were obtained at more than 95% purity (Fig 7B). Rubredoxin and Xylanase fusions led to an improvement in FP purification yield, with the rubredoxin fusion displaying the best purification yield at approximately 1.5 mg/L (Fig 7C). In contrast, T4L and PGS fusions decreased the observed yield, and did not improve the stability of FP (Fig 7C, 7D). Although the insertion of flavodoxin did not affect the purification yield of FP, both rubredoxin and flavodoxin fusions augmented the stability of FP with a Tm of 58 and 57^o^C, respectively. Overall, the rubredoxin fusion with FP displayed the best improvement in purification yield and stability compared to FP with a bRIL insertion.

**Fig 8:**
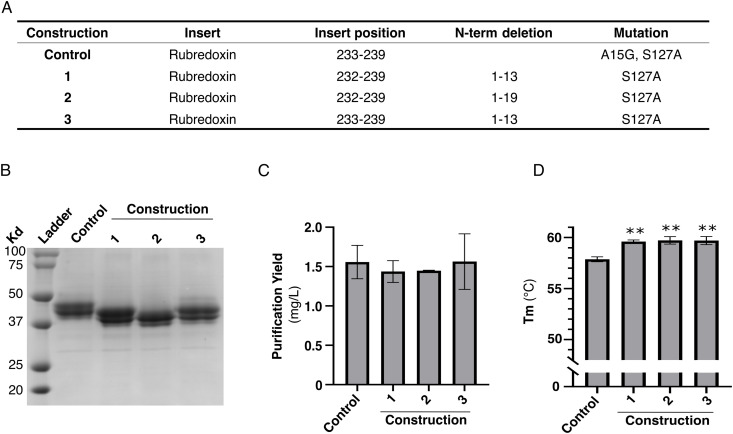
Optimized FP constructs. **(A)** Description of stable constructs. **(B)** Coomassie-stained 12% polyacrylamide gel, (C) purification yield and (D) melting temperature (Tm) of IMAC eluate of indicated FP constructs. The data are expressed as the means ± SEM of 3 independent experiments. Statistical significance is determined by one-way ANOVA followed by a Dunnett post-hoc analysis. ** p < 0.01.

Finally, we took advantage of the modifications, improving FP purification yield and stability, and opted to combine a few of the most effective ones (Fig 8). Although we found that N-terminal and C-terminal truncations display similar purification yield and stability compared to the untruncated receptor, removal of a flexible domain of the receptor could potentially prevent aggregation at the high concentration required for crystallography and cryoEM, as well as promote the nucleation process in crystallography. Thus, we have selected two of the least disrupting truncations, deletions of the N-terminus at residues 13 and 19, to assess their impact on FP constructs’ purification yield and stability, combining optimal fusions and mutations. We present the results of three new constructs that improve the rubredoxin fusion found in Fig 7. In addition to the N-terminal truncations, these constructs display rubredoxin insertion between residues 232–239 or 233–239 of FP ICL3, and the mutation S127A (Fig 8A). We successfully purified these constructs at a purity of more than 95% (Fig 8B). The purification yield was 1.5 mg/L for all the constructs, a result similar to the rubredoxin fusion of Fig 7 (control in Fig 8). In contrast, they displayed a 2^o^C improvement of their Tm, suggesting that combining optimal small domain insertions, mutations, and N-terminal truncation leads to the stabilization of the constructs. Together, we showed that we were able to improve the purification yield of the FP-cloprostenol complex by approximately threefold and the stability of the micelles-containing receptor by 9^o^C, leading to a protein preparation suitable for further structural biology studies.

## Discussion

GPCRs are inherently unstable *in vitro,* leading to receptor aggregation at concentrations used for structural biology. Here, we reported the optimization of the protein sequence of the FP-cloprostenol complex to obtain a stable and monodispersed purified receptor using approaches such as N- and C-terminal truncation, mutations, and the insertion of small soluble domains. Truncation of the unstructured N- and C-terminus is an interesting strategy in structural biology to reduce the probability of interfering with crystal contact and to improve diffraction, preventing receptor aggregation at high concentrations. The C-terminal domain of GPCRs is also important for receptor desensitization in cells. It is the target of many post-translational modifications, such as phosphorylation by protein kinases that induce the recruitment of ßarrestins, which act as scaffolding effectors for cellular desensitization machinery [30]. Truncation of the C-terminus of many GPCRs may increase the receptor purification yield by blocking cell surface receptor engagement of ßarrestins, and is a strategy that has been used for GPCRs optimization for structural biology [16,19,31,32]. Here, truncation of FP’s C-terminal domain did not affect receptor cell surface expression and *in vitro* stability, but rather generally slightly reduced the purification yield. This correlates with previous reports showing little or no FP coupling to ßarrestins in cells and points to distinct cellular regulation mechanisms of the receptor [3,33,34]. The absence of ßarrestins coupling has already been reported for EP3, another prostaglandin receptor [33,35]. Interestingly, we could not truncate the C-terminus of FP shorter than residue 324, located immediately before the two cysteines at position 322–323, without major effects on receptor stability. Many cysteine residues at the end of helix 8 of GPCRs are palmitoylated. This post-translational modification serves as a membrane anchor for helix 8 and is involved in receptor folding and trafficking [36]. Although palmitoylation of FP has never been described before, our findings suggest the critical importance of helix 8 between residues 308–323 for FP stability *in vitro*. Interestingly, cysteine 323 points toward the membrane in the FP cryoEM structure, suggests that the C-terminus of helix 8 could be palmitoylated and that the palmitoyl-cysteines could play an important role in FP stability. Another efficient strategy for stabilizing GPCRs is the introduction of mutations, and single point mutations have been shown to stabilize GPCRs for crystallization. For example, replacement of S91 by a lysine leads to a significant thermostabilization of the adenosine 2A receptor [37]. Similarly, the introduction of two mutations enabled the crystallization of the serotonin 2A receptor [32]. In our study, replacement of the membrane-exposed polar serine 127 by alanine, a closely related hydrophobic amino acid, probably tightens the hydrophobic bonds of transmembrane helix 3 with the micelles, leading to the increase of FP thermostability *in vitro*. Additional thermostabilizing mutations could be identified from the agonist-bound FP cryoEM structures [ 14,15]. These mutations could potentially enable the study of other FP ligands with different efficacies.

Insertion of small soluble domains is one of the most important modifications present in many of the GPCR structures. Here, we used the small soluble domain bRIL to screen for the optimal ICL3 insertion site. We then used the best site to screen for other small soluble domains, as they were designed initially with similar geometrical requirements for GPCR loop insertion [18]. However, there are different structural constraints among these domains. For example, the N-terminus of bRIL is an alpha helix that dictates its orientation within the fusion protein relative to the receptor. Thus, it is possible that the insertion site would differ slightly among the small soluble domains and re-optimization could be performed for each insert after the initial screen. Alternatively, the introduction of a glycine-serine linker on each side of the insert has already been used to circumvent this challenge [17].

The receptor engineering strategies employed in our study are applicable to the two main structural biology approaches used to solve GPCR structures at high-resolution: cryoEM and X-ray crystallography. The typical concentrations of purified GPCRs needed for these methods are similar, ranging from 2−5 and 20−30 mg/ml for cryoEM and X-ray crystallography, respectively. Although the protein concentration required for cryoEM is lower, both approaches require a similar amount of initial receptors due to the difficulty in cryoEM to obtain optimal vitrification conditions. Thus, a minimum purification yield of 0.3–0.5 mg/L of stable receptors is required for screening and collecting multiple EM grids, as well as for initial screens in X-ray crystallography [24]. Although cryoEM typically requires less receptor modification, most structures are agonist-bound due to the necessity to co-purify the receptor in a complex with the heterotrimeric G proteins and nanobodies to respect the mass limitation and circumvent the particle orientation problem. The presence of heterotrimeric G protein would likely shift the receptor equilibrium toward an activated state, displaying a structure with a higher affinity for agonists. Thus, obtaining an antagonist-bound cryoEM co-structure remains a challenge [38]. To date, X-ray crystallography has provided a greater diversity of co-structures with ligands of different efficacy due to the protein optimization effort to obtain receptor crystals [26]. Generally, the isolation of antagonist-receptor complexes within the prostaglandin receptor subfamily is notoriously challenging. The only available antagonist-bound co-structure is for the PGE_2_ type 4 receptor [21]. Similarly, FP is not stable in its apo form and in complex with an antagonist *in vitro*. Thus, we used the agonist cloprostenol as a stabilizing tool to obtain initial IMAC eluates of FP. It is possible that the impact of FP modifications would be specific for agonists, as antagonist co-purification with our optimal FP constructs did not yet yield stable IMAC eluates. Thus, solving the structure of antagonist-bound FP using cryoEM or X-ray crystallography approaches would require further receptor optimization. As such, our optimized FP constructs display a stability and purification yield well within structural biology working parameters and constitute a good initial framework for further receptor optimization.

## Supporting information

S1 File Raw images. (PDF)

S2 Fig Flow chart of the steps leading to FP purification by IMAC and characterization by microscale fluorescence thermal assay. Cloprostenol concentration (µM) and molar ratio (cloprostenol/FP) at each step of FP purification are indicated.
(TIF)

S3 Table Expression of the FP constructs in *Sf*9 cells. Cell expression was monitored by flow cytometry as previously described [24]. Briefly, 10 µL of infected cell culture grown during 48h were mixed with 10 µL of antibody solution (Tris Buffered Saline (TBS) supplemented with 11 µg/mL Monoclonal anti-FLAG M2-FITC antibodies (Sigma), 10 µg/mL eBioscience™ 7-Aminoactinomycin D (7-AAD) viability staining solution (Invitrogen) and 4% Bovine Serum Albumin). For total expression, antibody solution was supplemented with 0.3% Triton™ X-100 (Sigma). The mix was incubated at RT protected from light for 20 minutes. 180 µL of TBS were added, and fluorescence was measured using Guava^®^ EasyCyte™ mini cytometer (Cytek Bioscience Inc.) at 680 nm (red) for 7-AAD and 525 nm (green) for FITC. The percentage of expressing cells was determined by comparing FITC fluorescence of negative control (non-infected cells), with samples. The data are represented as the means ± SEM of 3 independent experiments.
(PDF)

## References

[1] Santos R, Ursu O, Gaulton A, Bento AP, Donadi RS, Bologa CG, et al. A comprehensive map of molecular drug targets. Nat Rev Drug Discov. 2017;16(1):19–34. doi: 10.1038/nrd.2016.230 27910877 PMC6314433

[2] Sriram K, Insel PA. G Protein-Coupled Receptors as Targets for Approved Drugs: How Many Targets and How Many Drugs? Mol Pharmacol. 2018;93(4):251–8. doi: 10.1124/mol.117.111062 29298813 PMC5820538

[3] Sedki D, Cho A, Cao Y, Nikolajev L, Atmuri NDP, Lubell WD, et al. Prostaglandin F2α and angiotensin II type 1 receptors exhibit differential cognate G protein coupling regulation. J Biol Chem. 2022;298(9):102294. doi: 10.1016/j.jbc.2022.102294 35872018 PMC9418914

[4] Hirata T, Narumiya S. Prostanoid receptors. Chem Rev. 2011;111(10):6209–30. doi: 10.1021/cr200010h 21819041

[5] Sugimoto Y, Inazumi T, Tsuchiya S. Roles of prostaglandin receptors in female reproduction. J Biochem. 2015;157(2):73–80. doi: 10.1093/jb/mvu081 25480981

[6] Mohan S, Koller EJ, Fazal JA, De Oliveria G, Pawlowicz AI, Doré S. Genetic Deletion of PGF2α-FP Receptor Exacerbates Brain Injury Following Experimental Intracerebral Hemorrhage. Front Neurosci. 2018;12:556. doi: 10.3389/fnins.2018.00556 30233287 PMC6134069

[7] Yu Y, Lucitt MB, Stubbe J, Cheng Y, Friis UG, Hansen PB, et al. Prostaglandin F2α elevates blood pressure and promotes atherosclerosis. Proc Natl Acad Sci U S A. 2009;106(19):7985–90. doi: 10.1073/pnas.0811834106 PMC2673134 19416858

[8] Sharif NA, Odani-Kawabata N, Lu F, Pinchuk L. FP and EP2 prostanoid receptor agonist drugs and aqueous humor outflow devices for treating ocular hypertension and glaucoma. Exp Eye Res. 2023;229:109415. doi: 10.1016/j.exer.2023.109415 36803996

[9] Beck H, Thaler T, Meibom D, Meininghaus M, Jörißen H, Dietz L, et al. Potent and Selective Human Prostaglandin F (FP) Receptor Antagonist (BAY-6672) for the Treatment of Idiopathic Pulmonary Fibrosis (IPF). J Med Chem. 2020;63(20):11639–62. doi: 10.1021/acs.jmedchem.0c00834 32969660

[10] Sharif NA, Klimko PG. Prostaglandin FP receptor antagonists: discovery, pharmacological characterization and therapeutic utility. Br J Pharmacol. 2019;176(8):1059–78. doi: 10.1111/bph.14335 29679483 PMC6451070

[11] Goupil E, Tassy D, Bourguet C, Quiniou C, Wisehart V, Pétrin D, et al. A novel biased allosteric compound inhibitor of parturition selectively impedes the prostaglandin F2alpha-mediated Rho/ROCK signaling pathway. J Biol Chem. 2010;285(33):25624–36. doi: 10.1074/jbc.M110.115196 20551320 PMC2919126

[12] Coler BS, Shynlova O, Boros-Rausch A, Lye S, McCartney S, Leimert KB, et al. Landscape of Preterm Birth Therapeutics and a Path Forward. J Clin Med. 2021;10(13):2912. doi: 10.3390/jcm10132912 34209869 PMC8268657

[13] Klimko PG, Sharif NA. Discovery, characterization and clinical utility of prostaglandin agonists for the treatment of glaucoma. Br J Pharmacol. 2019;176(8):1051–8. doi: 10.1111/bph.14327 29665040 PMC6451111

[14] Wu C, Xu Y, He Q, Li D, Duan J, Li C, et al. Ligand-induced activation and G protein coupling of prostaglandin F2α receptor. Nat Commun. 2023;14(1):2668. doi: 10.1038/s41467-023-38411-x 37160891 PMC10169810

[15] Lv X, Gao K, Nie J, Zhang X, Zhang S, Ren Y, et al. Structures of human prostaglandin F2α receptor reveal the mechanism of ligand and G protein selectivity. Nat Commun. 2023;14(1):8136. doi: 10.1038/s41467-023-43922-8 38065938 PMC10709307

[16] Audet M, Stevens RC. Emerging structural biology of lipid G protein-coupled receptors. Protein Sci. 2019;28(2):292–304. doi: 10.1002/pro.3509 30239054 PMC6319753

[17] Audet M, White KL, Breton B, Zarzycka B, Han GW, Lu Y, et al. Crystal structure of misoprostol bound to the labor inducer prostaglandin E2 receptor. Nat Chem Biol. 2019;15(1):11–7. doi: 10.1038/s41589-018-0160-y 30510194 PMC6289721

[18] Chun E, Thompson AA, Liu W, Roth CB, Griffith MT, Katritch V, et al. Fusion partner toolchest for the stabilization and crystallization of G protein-coupled receptors. Structure. 2012;20(6):967–76. doi: 10.1016/j.str.2012.04.010 22681902 PMC3375611

[19] Xiang J, Chun E, Liu C, Jing L, Al-Sahouri Z, Zhu L, et al. Successful Strategies to Determine High-Resolution Structures of GPCRs. Trends Pharmacol Sci. 2016;37(12):1055–69. doi: 10.1016/j.tips.2016.09.009 27726881

[20] Zhang K, Wu H, Hoppe N, Manglik A, Cheng Y. Fusion protein strategies for cryo-EM study of G protein-coupled receptors. Nat Commun. 2022;13(1):4366. doi: 10.1038/s41467-022-32125-2 35902590 PMC9334595

[21] Toyoda Y, Morimoto K, Suno R, Horita S, Yamashita K, Hirata K, et al. Ligand binding to human prostaglandin E receptor EP4 at the lipid-bilayer interface. Nat Chem Biol. 2019;15(1):18–26. doi: 10.1038/s41589-018-0131-3 30510193

[22] Namkung Y, Radresa O, Armando S, Devost D, Beautrait A, Le Gouill C, et al. Quantifying biased signaling in GPCRs using BRET-based biosensors. Methods. 2016;92:5–10. doi: 10.1016/j.ymeth.2015.04.010 25890247

[23] Namkung Y, LeGouill C, Kumar S, Cao Y, Teixeira LB, Lukasheva V, et al. Functional selectivity profiling of the angiotensin II type 1 receptor using pathway-wide BRET signaling sensors. Sci Signal. 2018;11(559):eaat1631. doi: 10.1126/scisignal.aat1631 30514808

[24] Audet M, Villers K, Velasquez J, Chu M, Hanson C, Stevens RC. Small-scale approach for precrystallization screening in GPCR X-ray crystallography. Nat Protoc. 2020;15(1):144–60. doi: 10.1038/s41596-019-0259-y 31784719

[25] Alexandrov AI, Mileni M, Chien EYT, Hanson MA, Stevens RC. Microscale fluorescent thermal stability assay for membrane proteins. Structure. 2008;16(3):351–9. doi: 10.1016/j.str.2008.02.004 18334210

[26] Pándy-Szekeres G, Munk C, Tsonkov TM, Mordalski S, Harpsøe K, Hauser AS, et al. GPCRdb in 2018: adding GPCR structure models and ligands. Nucleic Acids Res. 2018;46(D1):D440–6. doi: 10.1093/nar/gkx1109 29155946 PMC5753179

[27] Wacker D, Fenalti G, Brown MA, Katritch V, Abagyan R, Cherezov V, et al. Conserved binding mode of human beta2 adrenergic receptor inverse agonists and antagonist revealed by X-ray crystallography. J Am Chem Soc. 2010;132(33):11443–5. doi: 10.1021/ja105108q 20669948 PMC2923663

[28] Morimoto K, Suno R, Hotta Y, Yamashita K, Hirata K, Yamamoto M, et al. Crystal structure of the endogenous agonist-bound prostanoid receptor EP3. Nat Chem Biol. 2019;15(1):8–10. doi: 10.1038/s41589-018-0171-8 30510192

[29] Thorsen TS, Matt R, Weis WI, Kobilka BK. Modified T4 Lysozyme Fusion Proteins Facilitate G Protein-Coupled Receptor Crystallogenesis. Structure. 2014;22(11):1657–64. doi: 10.1016/j.str.2014.08.022 25450769 PMC4408211

[30] Gurevich VV, Gurevich EV. GPCR Signaling Regulation: The Role of GRKs and Arrestins. Front Pharmacol. 2019;10:125. doi: 10.3389/fphar.2019.00125 30837883 PMC6389790

[31] Wingler LM, McMahon C, Staus DP, Lefkowitz RJ, Kruse AC. Distinctive Activation Mechanism for Angiotensin Receptor Revealed by a Synthetic Nanobody. Cell. 2019;176(3):479-490.e12. doi: 10.1016/j.cell.2018.12.006 30639100 PMC6367718

[32] Kimura KT, Asada H, Inoue A, Kadji FMN, Im D, Mori C, et al. Structures of the 5-HT2A receptor in complex with the antipsychotics risperidone and zotepine. Nat Struct Mol Biol. 2019;26(2):121–8. doi: 10.1038/s41594-018-0180-z 30723326

[33] Avet C, Mancini A, Breton B, Le Gouill C, Hauser AS, Normand C, et al. Effector membrane translocation biosensors reveal G protein and βarrestin coupling profiles of 100 therapeutically relevant GPCRs. Elife. 2022;11:e74101. doi: 10.7554/eLife.74101 35302493 PMC9005190

[34] Goupil E, Wisehart V, Khoury E, Zimmerman B, Jaffal S, Hébert TE, et al. Biasing the prostaglandin F2α receptor responses toward EGFR-dependent transactivation of MAPK. Mol Endocrinol. 2012;26(7):1189–202. doi: 10.1210/me.2011-1245 22638073 PMC5416999

[35] Normand C, Breton B, Salze M, Barbeau E, Mancini A, Audet M. A systematic analysis of prostaglandin E2 type 3 receptor isoform signaling reveals isoform- and species-dependent L798106 Gαz-biased agonist responses. Eur J Pharmacol. 2022;927:175043. doi: 10.1016/j.ejphar.2022.175043 35598847

[36] Patwardhan A, Cheng N, Trejo J. Post-Translational Modifications of G Protein-Coupled Receptors Control Cellular Signaling Dynamics in Space and Time. Pharmacol Rev. 2021;73(1):120–51. doi: 10.1124/pharmrev.120.000082 33268549 PMC7736832

[37] Claff T, Klapschinski TA, Tiruttani Subhramanyam UK, Vaaßen VJ, Schlegel JG, Vielmuth C, et al. Single Stabilizing Point Mutation Enables High-Resolution Co-Crystal Structures of the Adenosine A2A Receptor with Preladenant Conjugates. Angew Chem Int Ed Engl. 2022;61(22):e202115545. doi: 10.1002/anie.202115545 35174942 PMC9310709

[38] Xu J, Chen G, Wang H, Cao S, Heng J, Deupi X, et al. Calcineurin-fusion facilitates cryo-EM structure determination of a Family A GPCR. Proc Natl Acad Sci U S A. 2024;121(48):e2414544121. doi: 10.1073/pnas.2414544121 39565314 PMC11621825

