# Supplementary figures and images for: Optimization of the prostaglandin F2α receptor for structural biology
Source: PLoS One. 2025 Jul 18;20(7):e0320114. doi: 10.1371/journal.pone.0320114 (PMC12273924; doi:10.1371/journal.pone.0320114)

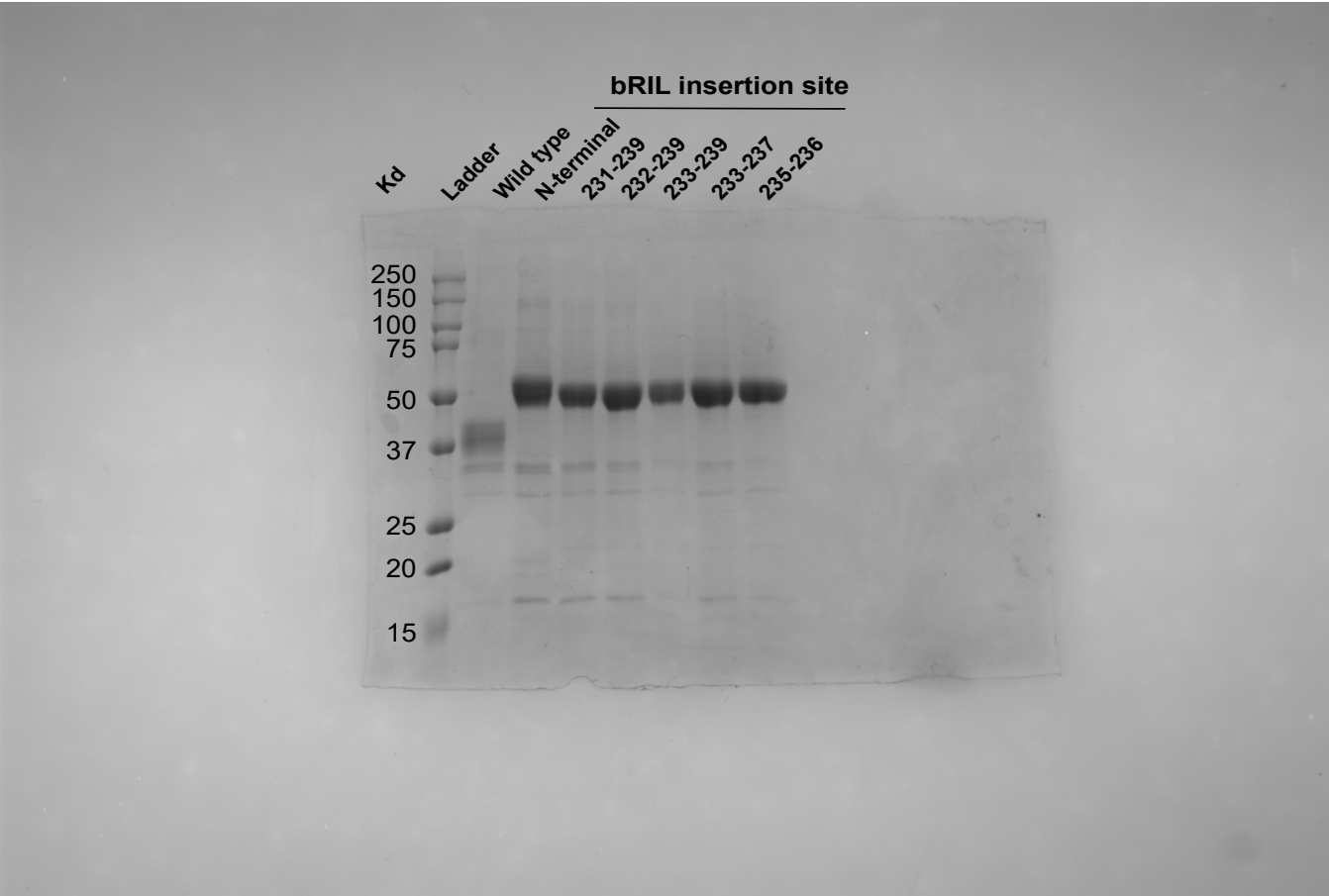

Fig 2B

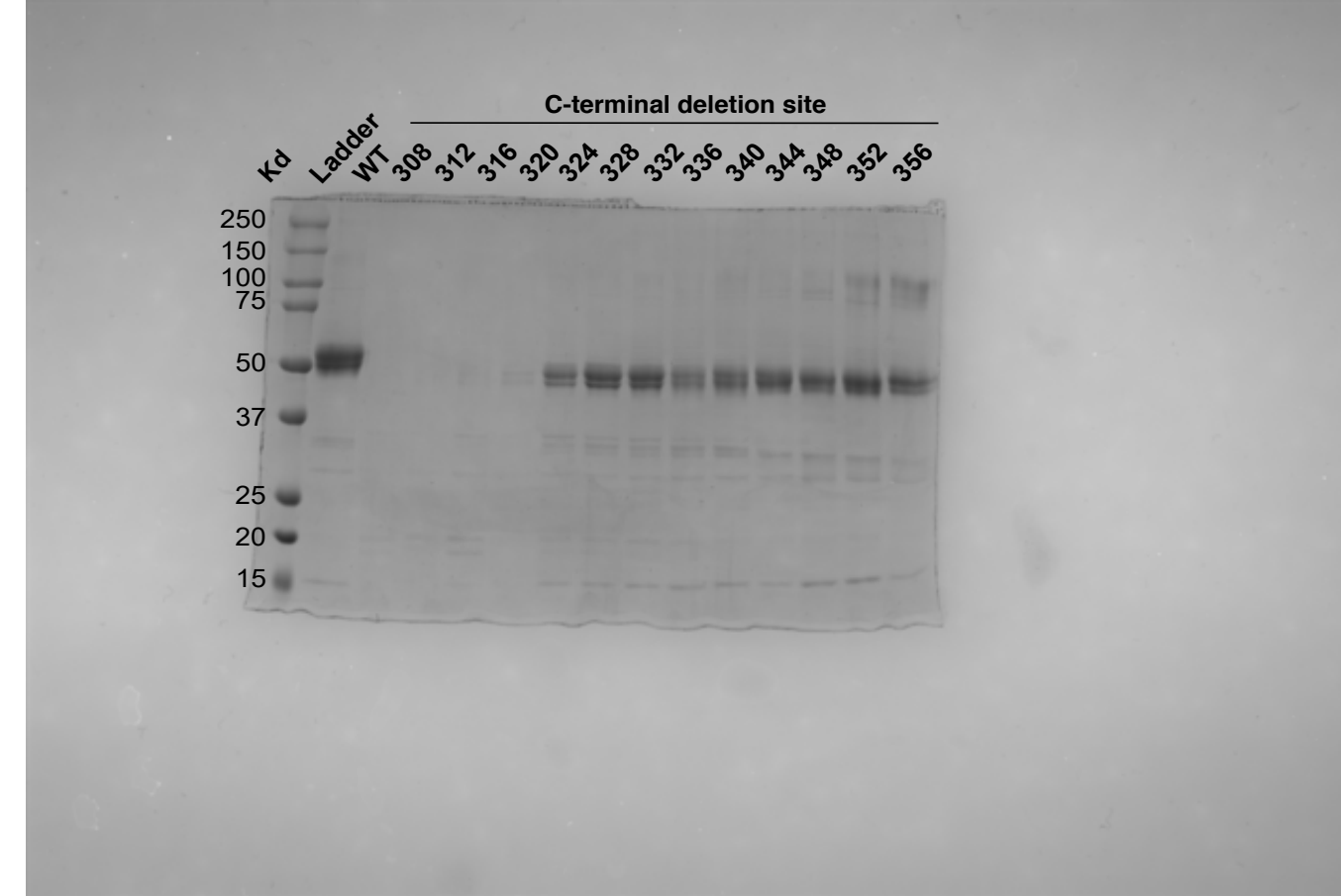

Fig 4B

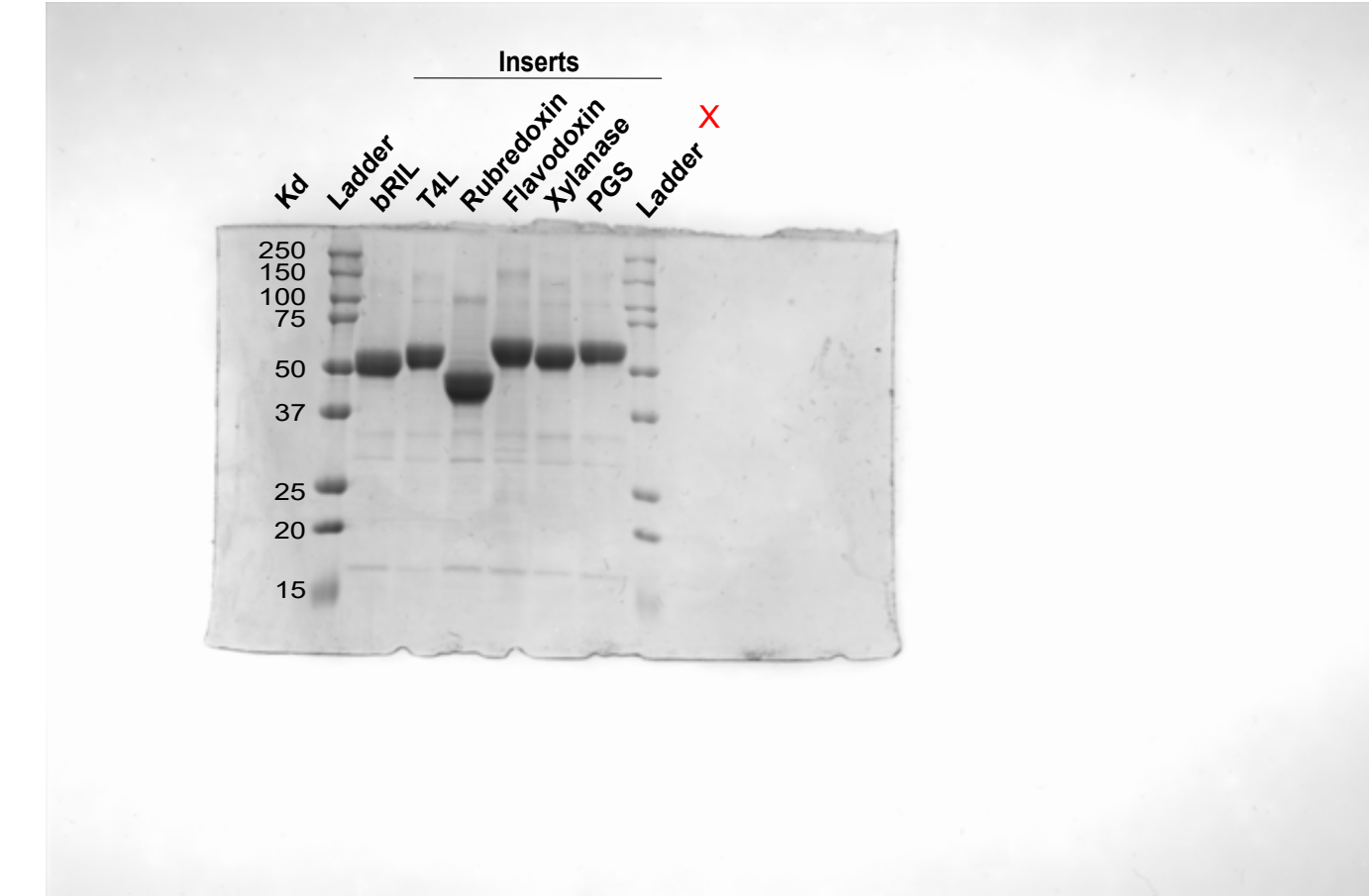

Fig 7B

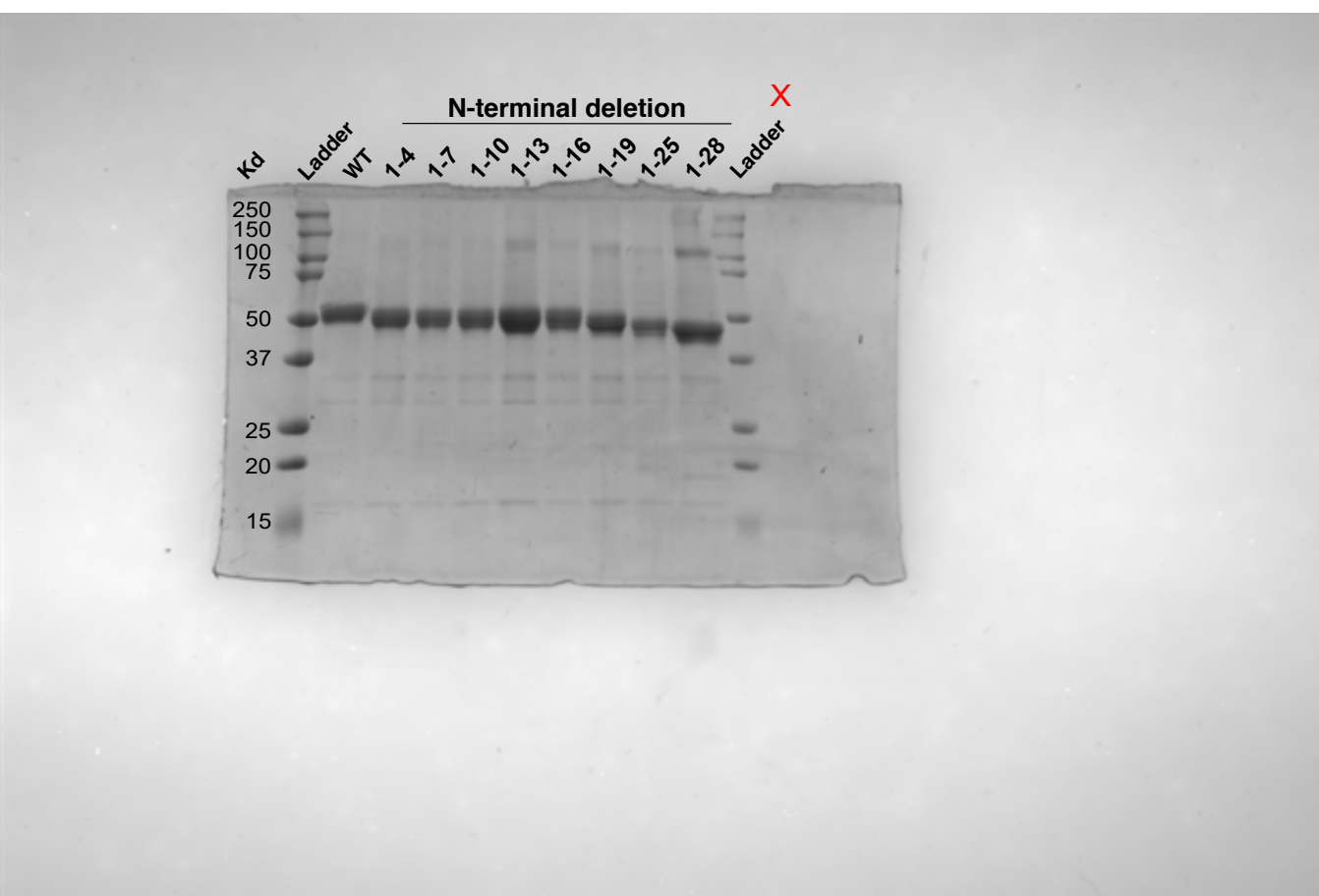

Fig 3B

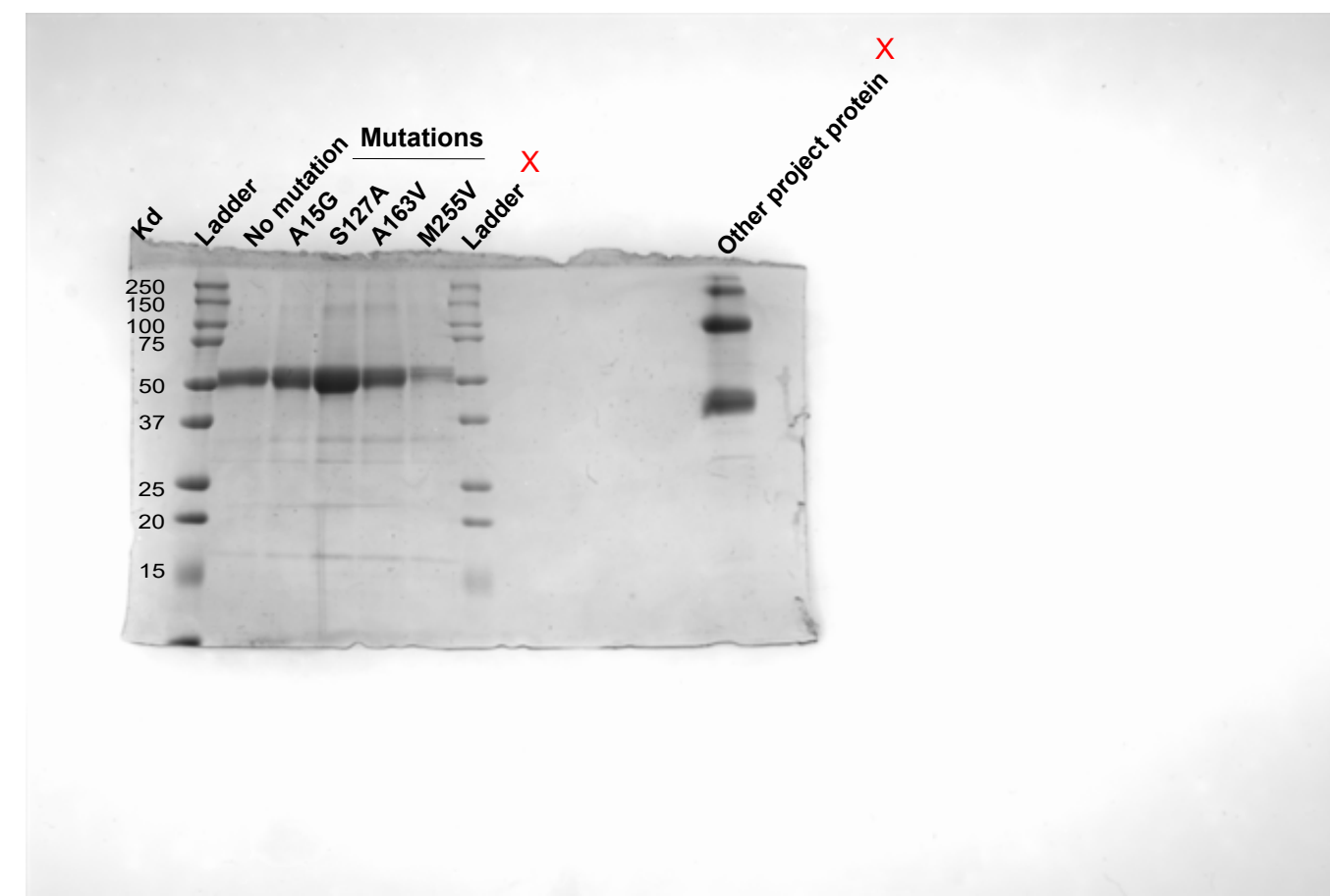

Fig 5B

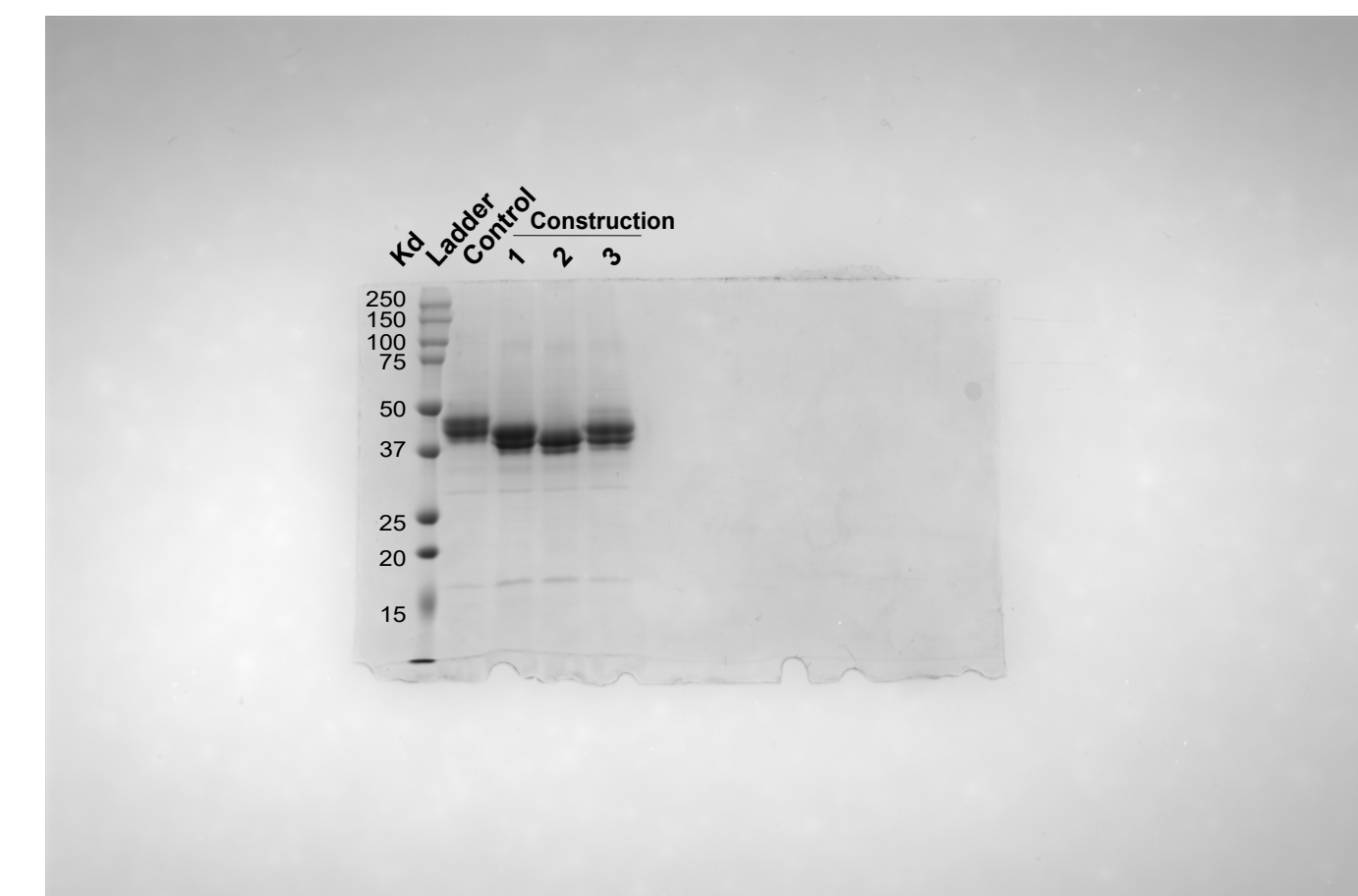

Fig 8B

Supplement: S1 File — (PDF) [file pone.0320114.s001.pdf]

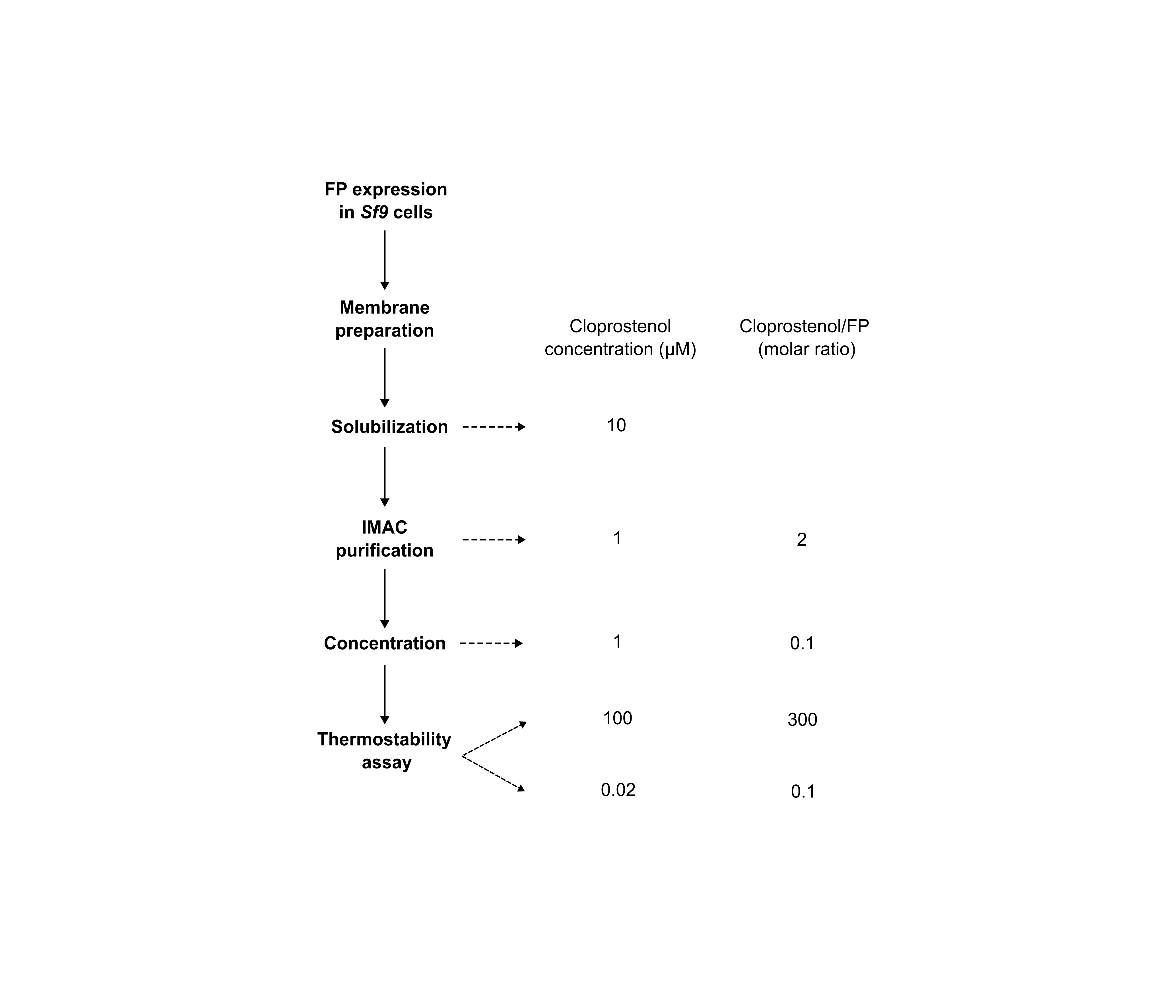

Supplement: S2 Fig — Cloprostenol concentration (µM) and molar ratio (cloprostenol/FP) at each step of FP purification are indicated. (TIF) [file pone.0320114.s002.tif]
