# Supplementary material for: Optimization of the prostaglandin F2α receptor for structural biology
Source: PLoS One. 2025 Jul 18;20(7):e0320114. doi: 10.1371/journal.pone.0320114 (PMC12273924; doi:10.1371/journal.pone.0320114)
Supplement: S3 Table — Cell expression was monitored by flow cytometry as previously described [24]. Briefly, 10 µL of infected cell culture grown during 48h were mixed with 10 µL of antibody solution (Tris Buffered Saline (TBS) supplemented with 11 µg/mL Monoclonal anti-FLAG M2-FITC antibodies (Sigma), 10 µg/mL eBioscience™ 7-Aminoactinomycin D (7-AAD) viability staining solution (Invitrogen) and 4% Bovine Serum Albumin). For total expression, antibody solution was supplemented with 0.3% Triton™ X-100 (Sigma). The mix was incubated at RT protected from light for 20 minutes. 180 µL of TBS were added, and fluorescence was measured using Guava® EasyCyte™ mini cytometer (Cytek Bioscience Inc.) at 680 nm (red) for 7-AAD and 525 nm (green) for FITC. The percentage of expressing cells was determined by comparing FITC fluorescence of negative control (non-infected cells), with samples. The data are represented as the means ± SEM of 3 independent experiments. (PDF) [file pone.0320114.s003.pdf]

			% of cell expression			
			Surface		Total	
**Insertion**	**Deletion**	**Mutations**	**Mean**	**SEM**	**Mean**	**SEM**
none (WT)			87	10	81	7
bRIL N-terminal			92	7	79	4
bRIL 231-239			92	6	81	6
bRIL 232-239			95	4	85	6
bRIL 233-239			96	3	89	6
bRIL 233-237			96	3	91	6
bRIL 235-236			97	2	93	4
bRIL 232-239	1-4		95	4	87	7
bRIL 232-239	1-7		95	4	87	6
bRIL 232-239	1-10		95	4	86	7
bRIL 232-239	1-13		95	4	90	5
bRIL 232-239	1-16		96	3	88	3
bRIL 232-239	1-19		95	5	88	6
bRIL 232-239	1-25		94	4	84	9
bRIL 232-239	1-28		95	3	87	7
bRIL N-terminal	308-end		84	6	94	1
bRIL N-terminal	312-end		83	7	96	1
bRIL N-terminal	316-end		74	12	95	1
bRIL N-terminal	320-end		65	10	96	1
bRIL N-terminal	324-end		87	5	86	6
bRIL N-terminal	328-end		88	8	76	9
bRIL N-terminal	332-end		91	3	81	2
bRIL N-terminal	336-end		89	5	83	4
bRIL N-terminal	340-end		88	3	87	3
bRIL N-terminal	344-end		92	3	87	4
bRIL N-terminal	348-end		86	5	82	5
bRIL N-terminal	352-end		91	3	78	6
bRIL N-terminal	356-end		93	2	78	3
bRIL 232-239		A15G	96	1	78	2
bRIL 232-239		S127A	96	2	73	6
bRIL 232-239		A163A	95	4	79	4
bRIL 232-239		M255V	93	2	83	4
bRIL 233-239		A15G, S127A	97	2	89	5
T4L 233-239		A15G, S127A	95	4	97	1
Rubredoxin 233-239		A15G, S127A	97	2	96	2
Flavodoxin 233-239		A15G, S127A	94	4	95	4
Xylanase 233-239		A15G, S127A	97	3	95	1
PGS 233-239		A15G, S127A	96	4	95	3
Rubredoxin 232-239	1-13	S127A	94	3	93	3
Rubredoxin 232-239	1-19	S127A	93	2	90	1
Rubredoxin 233-239	1-13	S127A	94	2	92	1
